# 

**Published:** 1900-10-27

**Authors:** 


### NOTICE TO CORRESPONDENTS.

All MSS., Letters, Books for Review, and other matters intended for the Editor should be addressed The Editor, "The Hospital," The Hospital Building, 28 & 29 Southampton Street, Strand, W.C.

The Editor cannot undertake to return rejected MS., even when accompanied by stamped directed envelope.

**Notice to Advertisers and Subscribers.**

All Advertisements, Orders for Copies of The Hospital, and Business Communications should be addressed to **THE MANAGER (not to the Editor),** The Hospital, The Hospital Building, 28 & 29 Southampton Street, Strand, London, W.C.

The Cost of Subscription, post free, is as follows:—Per week, 2½d.; per quarter, 2s. 8d.; per half-year, 5s. 3d.; per annum, 10s. 6d. Foreign.—Per annum, 15s. 2d., payable, in all cases, in advance.

**NOTICE.—Vol. XXVIII. of "The Hospital" (April to September, 1900), in handsome cloth binding, will shortly be on sale at the Office of this Journal, price, 7s. 6d. Cases for binding the Numbers contained in this Volume 1s. 6d. Index to Vol. XXVIII., 2½d., post free.**

**The Monthly Part for November will be ready on November 1st. Price 6d., by post 8d.**

### BOOKS RECEIVED.

Love and Wyman.

"The Hospitals Inquiry." By G. M. Asher.

John Dutton, Chelmsford.

"Summary of the Reports of the District Medical Officers of Health in the Administrative County of Essex for 1899." By John C. Thresh, D.Sc., M.D., D.P.H.

Dean and Son.

"The Diverting History of John Gilpin." By William Cowper.
"Ten Little Boer Boys." By "Norman."

West, Newman and Co.

"Archives of Surgery." By Jonathan Hutchinson, LL.D., F.R.S.

The Sanitary Publishing Company.

"Essays on Consumption." By J. Edward Squire, M.D. Lond., D.P.H. Camb. With an Introduction by Sir William Broadbent, Bart. M.D., F.R.C.P.

Williams and Norgate.

Williams and Norgate's Book Circular.

### The Student of Medicine.

### APPOINTMENTS.

W. J. Adye, M.R.C.S.Eng., appointed Certifying Factory Surgeon for the Urban District of Bradford-on-Avon and the Civil Parishes of Monkton Farleigh, South Wroxall, Holt, Bradford Without, and Westwood in the Bradford Rural District. William C. Allardice, M.D.Glasg., appointed Certifying Factory Surgeon for Newcastle-under-Lyme. Percy S. Blaker, M.R.C.S., L.R.C.P., appointed Resident Medical Officer to the East London Hospital for Children, Shadwell. George Browne, M.D., M.Ch., R.U.I., appointed Certifying Factory Surgeon for the Ballynahinch District of County Down. W. F. Byford, M.R.C.S., L.R.C.P.Lond., appointed Certifying Factory Surgeon for the Ruthin District of the County of Denbigh. James Cook, M.B., appointed Medical Officer at Barcaldine, Queensland. George F. Dickinson, M.R.C.S.Lond., L.R.C.P.Lond., appointed Honorary Surgeon to the Warneford Hospital, Leamington. W. A. Forsyth, M.B., appointed Health Officer for the Shire of Mansfield, Victoria. Charles B. Gervis, M.D.Brux., M.R.C.S., L.R.C.P., appointed Public Vaccinator to the Seaford District of the Eastbourne Union. F. A. Hardy, M.B., M.S., appointed a

Clinical Assistant to the Chelsea Hospital for Women. R. A. L. Hill, M.R.C.S., L.R.C.P., appointed Surgeon to the Wimbledon Division of the Metropolitan Police. Guy Hollings, M.D., B.Ch., appointed a Clinical Assistant to the Chelsea Hospital for Women. J. E. Judson, M.R.C.S., L.R.C.P., appointed Junior Assistant Resident Medical Officer of the Manchester Workhouse, Crumpsall. Leslie Kingsford, M.R.C.S., L.R.C.P., appointed Medical Officer to the Casualty Department of the East London Hospital for Children. James B. Littlejohn, M.A., M.D., appointed Professor of Pathology and Bacteriology, Dunham Medical College, Chicago, Illinois, U.S.A. Arthur F. Perigal, M.B., Ch.B.Edin., appointed Resident House-Physician at the Brompton Hospital for Consumption and Diseases of the Chest. J. D. K. Scott, M.B., appointed Health Officer for the Borough of Queenscliff, Victoria. H. Carlile Sturdy, M.B.Durh., M.R.C.S., L.R.C.P., appointed House-Surgeon to the East London Hospital for Children, Shadwell. Philip C. Walker, M.D., D.P.H., appointed Assistant to the Medical Officer of Health of Paisley and Resident Medical Officer at the Borough Hospital for Infectious Diseases.

### SCRAPS AND GLEANINGS.

At a recent meeting of the Board of Management of the Swansea Hospital it was decided to apply to the Corporation for a supply of electricity for the laundry.

Through the death of Mrs. Warden, the Coventry and Warwickshire Hospital has benefited under the will of the late Mr. Warden to the extent of upwards of £1,000.

The recent parade in connection with the Brighton and Hove Friendly Societies produced a sum of £100, which has been distributed amongst seven Brighton hospitals and institutions.

During the past year 132 cases have been admitted to and treated in the Forfar Infirmary, being 40 fewer that the previous year. The total income for the year was £587 odd and the expenditure was £541 odd.

The estimated cost of the necessary improvements at Guisbrough Miners Hospital is £1,000. Against this the Committee have only £600 in hand, so, if the much-needed improvements are to be carried out, the generous help of the public is urgently needed.

In his speech at the recent opening of the class of surgery in Edinburgh University, Professor Chiene stated that, putting aside the foreign ambulances, more than 50 per cent. of those who served as medical men on the Boer side were taught in the Edinburgh school.

The receipts of the Metropolitan Hospital Saturday Fund from January 9th to September 8th from the various workshops and business houses have amounted to £8,889 odd, being a decrease, as compared with the corresponding period of last year, of £133. The expenditure since January reached £1,356 odd.

The honorary secretary and treasurer of the Cheltenham Hospital appeals in the local press for subscriptions to enable a balcony to be erected to Ward No. 9. The balcony that has been built to Ward No. 8 has been found of great use, and it is desired that No. 9 Ward should also have a balcony ready by next summer. £100 is in hand towards this object, and a further sum of £80 is required.

The foundation-stone of the new small-pox hospital for Croydon and Wimbledon was recently laid. The total quantity of land purchased in connection with this hospital is 56 acres, the price paid being £150 per acre. Seven acres of this land are required for the immediate purposes of the hospital, and three acres are to be sold to the Epsom Rural District Council. The hospital will contain accommodation for 46 beds and the nursing quarters will provide for 10 nurses.

The opening ceremony of the Aston Union Cottage Homes, Birmingham (which have been erected at a cost of between £50,000 and £60,000), was recently performed by Mr. James Evans (chairman of the Board of Guardians). In the course of his remarks Mr. Evans stated that at present there was accommodation for 250 children, but it was anticipated that ultimately 400 would be provided for. The buildings comprised 14 homes to accommodate 16 children each, two homes to accommodate 12 children each, a porter's lodge and receiving ward to accommodate 16 children, an infirmary to accommodate 24 patients, in addition to a church and school, workshops, swimming bath, &c.

### BOOKS FOR NURSES.

**Chavasse's Advice to a Wife,** 14th edition, by Dr. FANCOURT BARNES ... ... ... ... **2/6**

**Chavasse's Advice to a Mother,** 15th edition, by Dr. GEORGE CARPENTER... ... ... ... **2/6**

"No books of the kind in any way approach these two in usefulness, trustworthiness, and especially in friendliness and delicacy."—*Daily Mail.*

**Cuff's Lectures on Medicine to Nurses,** 3rd ed. **3/6**

**Domville's Manual for Hospital Nurses,** 8th ed. **2/6**

**Cullingworth's Monthly Nursing,** 4th edition.. **1/6**

**Hellier's Gynæcological Nursing** ... ... **1/6**

**Parkes' Elements of Health and Hygiene...** **3/6**

**Mayne's Short Medical Dictionary** ... ... **2/6**

LONDON: J. & A. CHURCHILL, 7 Great Marlborough Street.

*Second Edition Now Ready.*

### PRACTICAL NURSING.

By ISLA STEWART, Matron of St. Bartholomew's Hospital, London; and HERBERT E. CUFF, M.D., F.R.C.S., Medical Superintendent North-Eastern Fever Hospital, Tottenham, London.

With Diagrams. In 2 vols., crown 8vo. Vol. I., 3s. 6d. net.

"A most successful handbook for nurses, and should have a successful career."—*Hospital.*

"An eminently readable and practical handbook on nursing."—*Medical Review.*

WILLIAM BLACKWOOD AND SONS, Edinburgh and London.

Crown 8vo., over 300 pp., cloth, **2s.** net; post free, **2s. 4d.**

28 & 29 Southampton Street, Strand, W.C.

